# Case Report: Novel Biallelic Variants in the *COL18A1* Gene in a Chinese Family With Knobloch Syndrome

**DOI:** 10.3389/fneur.2022.853918

**Published:** 2022-05-26

**Authors:** Shuk Ching Chong, Yuet-Ping Yuen, Ye Cao, Sze-Shing Fan, Tak Yeung Leung, Emily K. Y. Chan, Xian Lun Zhu

**Affiliations:** ^1^Department of Paediatrics, Prince of Wales Hospital, The Chinese University of Hong Kong, Shatin, Hong Kong SAR, China; ^2^The Chinese University of Hong Kong-Baylor College of Medicine Joint Center of Medical Genetics, The Chinese University of Hong Kong, Shatin, Hong Kong SAR, China; ^3^Department of Obstetrics & Gynaecology, The Chinese University of Hong Kong, Shatin, Hong Kong SAR, China; ^4^Department of Pathology, Hong Kong Children's Hospital, Kowloon, Hong Kong SAR, China; ^5^Division of Neurosurgery, Department of Surgery, The Chinese University of Hong Kong, Shatin, Hong Kong SAR, China

**Keywords:** case report, Knobloch syndrome, COL18A1, collagenopathy, retinal detachment

## Abstract

Knobloch syndrome is a rare collagenopathy characterized by severe early onset myopia, retinal detachment, and occipital encephalocele with various additional manifestations due to biallelic changes in the *COL18A1* gene. Here we reported a Chinese family with two affected siblings presented with antenatal occipital encephalocele, infantile onset retinal detachment, and pronounced high myopia at early childhood. Quartet whole exome sequencing was performed in this family and identified that both siblings carried novel compound heterozygous variants in the *COL18A1* gene (NM_001379500.1): the maternally inherited variant c.1222-1G>A at the consensus acceptor splice site of intron 8, and the paternally inherited frameshift variant c.3931_3932delinsT p.(Gly1311Serfs^*^25) in the last exon. Both patients had successful surgical treatment for the occipital encephalocele soon after birth. They had normal neurocognitive outcome and good general conditions examined at the age of 7 years old for the elder sister and 4 years old for the younger brother. The younger brother developed infantile onset retinal detachment at 7 months of age while the sister had high myopia without signs of retinal detachment until 7 years old. This report expands the phenotype and genotype spectrum of Knobloch syndrome with antenatal and postnatal findings.

## Introduction

Collagenopathies are a diverse group of disorders due to defects in the collagen genes with clinically and genetically heterogeneity (1). Currently, 28 types of collagens are known to form important connective components of human tissue throughout the body (2). Besides this, collagens also participate in various biological processes as signaling molecules defining cellular shape and behavior. Therefore, these collagenopathies usually affect multiple organs with congenital or early age onset.

Knobloch syndrome [MIM 267750] firstly described by Knobloch and Layer (3), is a very rare collagenopathy caused by biallelic variants in the *COL18A1* gene (4). An updated literature search showed <100 patients or families with different types of mutations were reported. This syndrome is characterized with ocular and cranial abnormalities including remarkable high-grade early onset myopia, retinal detachment, and occipital encephalocele (5). Ocular features include childhood glaucoma, cataract, and lens subluxation (5–7). Fundus characteristics include collapsed vitreous, macular atrophy, and electroretinography showed cone-rod dysfunction in some of the reported cases (5). Occipital defects can present as encephalocele or only focal cutaneous defects. It is a high penetrance feature but not seen in some patients with physical examination (7). The majority of reported patients have normal intellectual ability, while additional findings including brain structure malformations, cognitive decline, epilepsy, developmental delay, delayed skin wound healing, and renal abnormalities were also reported (8–10). The spectrum of clinical variability is not completely known due to the small number of cases reported in the literature.

The *COL18A1* gene is located on chromosome 21q22.3. It encodes the alpha chain of type XVIII collagen which is a ubiquitous basement membrane (BM) proteoglycan with multiple functions. It has three distinct isoforms due to the two promoters and alternative splicing. They differ in lengths, tissue distribution, and N-terminal non-collagenous domain while the main component of the collagen XVIII protein structure is similar (11). The *COL18A1* protein also produce endostatin *via* proteolytic cleavage which is a signaling molecule known to inhibit the migration and proliferation of endothelial cells and suppress angiogenesis (12). Even though *COL18A1* is widely expressed, it is interesting that patients with mutations affecting all isoforms do not commonly have multiple abnormalities involving various organs other than the eye and posterior part of the skull. This suggested a particular contribution of collagen XVIII in ocular and skull development, including retinal structure formation and neural tube closure (5, 11). Aikio demonstrated that Collagen XVIII short isoform was critical for retinal vascularization in mice (13). As it is important to maintain the retinal structure, its dysfunction results in vitreoretinal degeneration and retinal detachment in severe cases. Other than encephalocele, patients with Knobloch syndrome could present with other extraocular manifestations such as end-stage renal failure at a young age (9) and delayed skin healing. Early-onset renal failure may result from basement membrane dysfunction, as animal studies show observation with raised creatinine and ultrastructural changes in basement membrane of kidney in *COL18A1* knockout mice (14, 15). The delayed skin healing would be associated with dysfunction of dermal-epidermal junction of the skin during wound healing (16).

Here the authors describe two siblings from a non-consanguineous Chinese family with two novel compound heterozygous variants in the *COL18A1* gene, suffering from antenatal encephalocele, ophthalmological manifestation, and retinal detachment at infancy with variable severity.

## Case Report

The index patient was a 7 year-old female, who was born at 38 weeks' gestation, with birth weight 3,010 g by elective cesarean section. Apgar score was 9 at 1 min and 10 at 5 min of life. Antenatal ultrasound scan at 20 weeks' gestation detected an occipital meningocele sized 14 mm × 11 mm × 11 mm, which later showed interval increased in size (Table 1). It was confirmed by fetal MRI at 36 weeks' gestation which showed an occipital meningocele measuring 11 mm × 27 mm × 16 mm. There was ventriculomegaly noted all along. Antenatal karyotyping and array comparative genomic hybridization (aCGH) of amniotic cells were normal. Postnatally, she was stable at birth and did not require mechanical ventilation. Her MRI brain showed a 20 mm × 18 mm × 25 mm cystic lesion at midline of occipital region while operation with excision of this meningocele was performed on day 6 of life. There was a small bony defect (<5 mm) with narrow pedicle connecting the lesion. The content of the meningocele was gel-like and no normal brain tissue. Her clinical condition was monitored regularly while she had no sign of raised intracranial pressure after surgery. At 1 year of age, she had another cystic lesion at the retrocerebellar region as shown on a follow-up MRI scan without other clinical signs. Posterior fossa craniotomy for cyst fenestration was performed at 16 months of age without any complications. She has no further intracranial cystic lesions developed and no hydrocephalus with generally normal development. She also had bilateral severe myopia since infancy requiring corrective glasses. Currently the severity of myopia was around −14.00D bilaterally. Retina on both eyes were thinning with indirect barrier laser treatment performed while no retinal detachment was noted so far. She can read at close proximity and write down her name in Chinese and English properly. The patients' parents reported her daily function was adjusted despite severe myopia. Her head circumference increased from 25th centile at birth to around 50th centile at 7 years old. Her development and cognitive assessment were normal at 7 years old.

**Table 1:** The core clinical features and comparison of these two siblings.

	**Older sister**	**Younger brother**
Age	7 years old	4 years old
Sex	Female	Male
Head circumference (cm, percentile)	51.5 cm, 75th	50.2 cm, 50th
Weight (kg, percentile)	22 kg, 50–75th	14 kg, 10–25th percentile
Height (cm, percentile)	115 cm, 10–25th percentile	94 cm, 10–25th percentile
**Ocular abnormalities**		
Myopia	Bilateral severe myopia (since infancy) (current: −14.50D bilaterally)	Bilateral severe myopia (very early onset) −14.5D/−15.5D
Aided Visual acuity	Aided Visual acuity 20/200 (right eye), 20/120 (left eye)	
Retina	Thinning retina on both eye No retinal detachment so far	Retinal detachment (infantile onset: 7 months old) -left eye: total -right eye: partial
Intraocular pressure	14.5 (right eye), 15 (left eye)	17 (left eye), 15 (right eye)
Axial length	22.3 mm	21.36 mm (left eye)
Fundus:	Degenerative retina with myopic fundus	Degenerative retina with myopic fundus
**Cranial abnormalities**		
Antenatal (at 20 weeks by US)	Occipital meningocele (14 × 11 × 11 mm)	Occipital meningocele (5 × 6 × 7 mm)
Antenatal (at 34–36 weeks gestation by MRI)	- Occipital meningocele (11 × 27 × 16 mm) -Ventriculomegaly	- Occipital meningocele (30 × 16 × 30 mm) -absence of inferior cerebellar vermis
Postnatal MRI	- Cystic lesion at midline of occipital region (20 × 18 × 25 mm) -small bony defect (<5 mm) with narrow pedicle connecting the lesion	- hypoplasia of inferior cerebellar vermis -dysgenesis of corpus callosum -small skull defect in the midline occipital bone with herniation of CSF-filled sac

**Figure 1:**
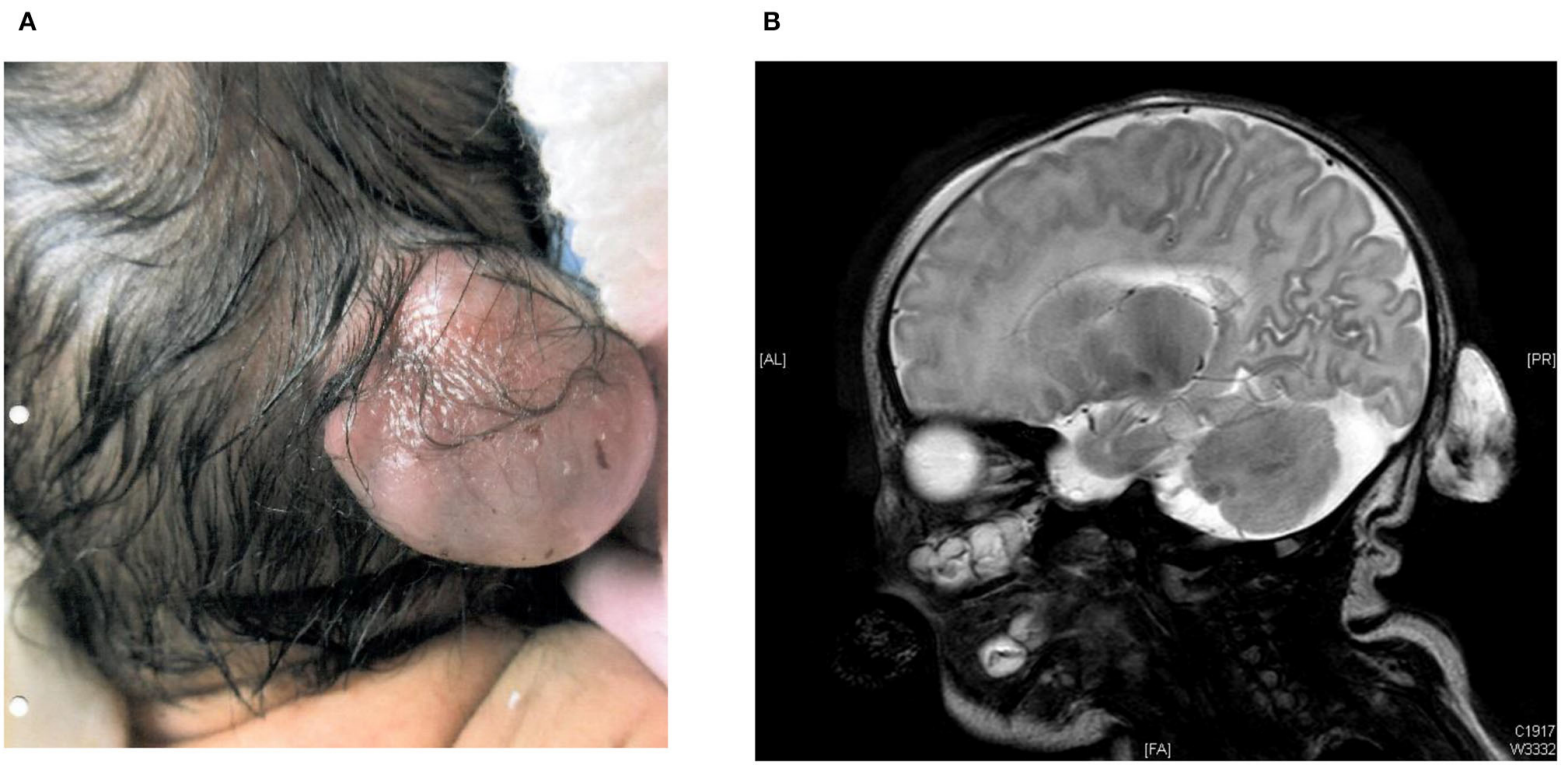
**(A)** 30 mm × 20 mm × 30 mm encephalocele in occipital region at birth in the younger brother. **(B)** MRI brain on day 2 of life from the younger brother.

The younger brother was currently 4 years old. He was born at 38 weeks gestation by elective Cesarean section with birth weight 3.07 kg. His Apgar score was 9 at one min and 9 at five min of life. Antenatal ultrasound scan detected a fetal occipital meningocele at 20 weeks' gestation, and its size was 5 mm × 6 mm × 7 mm (Table 1). Serial ultrasound scans and MRI scans showed the meningocele was growing bigger in size. The MRI scan at 34 weeks gestation showed the occipital meningocele measured 30 mm × 16 mm × 30 mm, and it detected an absence of inferior cerebellar vermis. The younger brother was born uneventfully. There was a 30 mm × 20 mm × 30 mm encephalocele seen at the occipital region, and it was cystic on palpation (Figure 1A). Clinically he was stable, and did not require mechanical ventilation. His brain MRI on day 2 of life showed hypoplasia of the inferior cerebellar vermis, dysgenesis of corpus callosum, a small skull defect in the midline occipital bone where there was the herniation of CSF-filled sac while the myelination was within normal limits. There was no definite brain tissue within the sac but evidence of meningeal and vascular contents attached to the cerebellum (Figure 1B). The repairment surgery with excision of meningocele was performed on day 5 of life without complications. His head circumference was growing on the 50th centile all along. He had very early onset bilateral severe myopia while total retinal detachment of left eye and partial retinal detachment over right eye were noted at 7 months old. He went through multiple eye surgeries including vitretomy with endolaser to retina, iridotomy, lensectomy for left eye and scleral buckling for right eye. Total retinal detachment of right eye was detected at the age of 1 year old, he had laser therapy to the right eye as well. His current myopia was −14.5D/-15.5D. His neurocognitive development was normal at 4 years old with normal gross motor and fine motor function. His parents reported his daily and social functions were normal.

## Genetic Test

**Figure 2:**
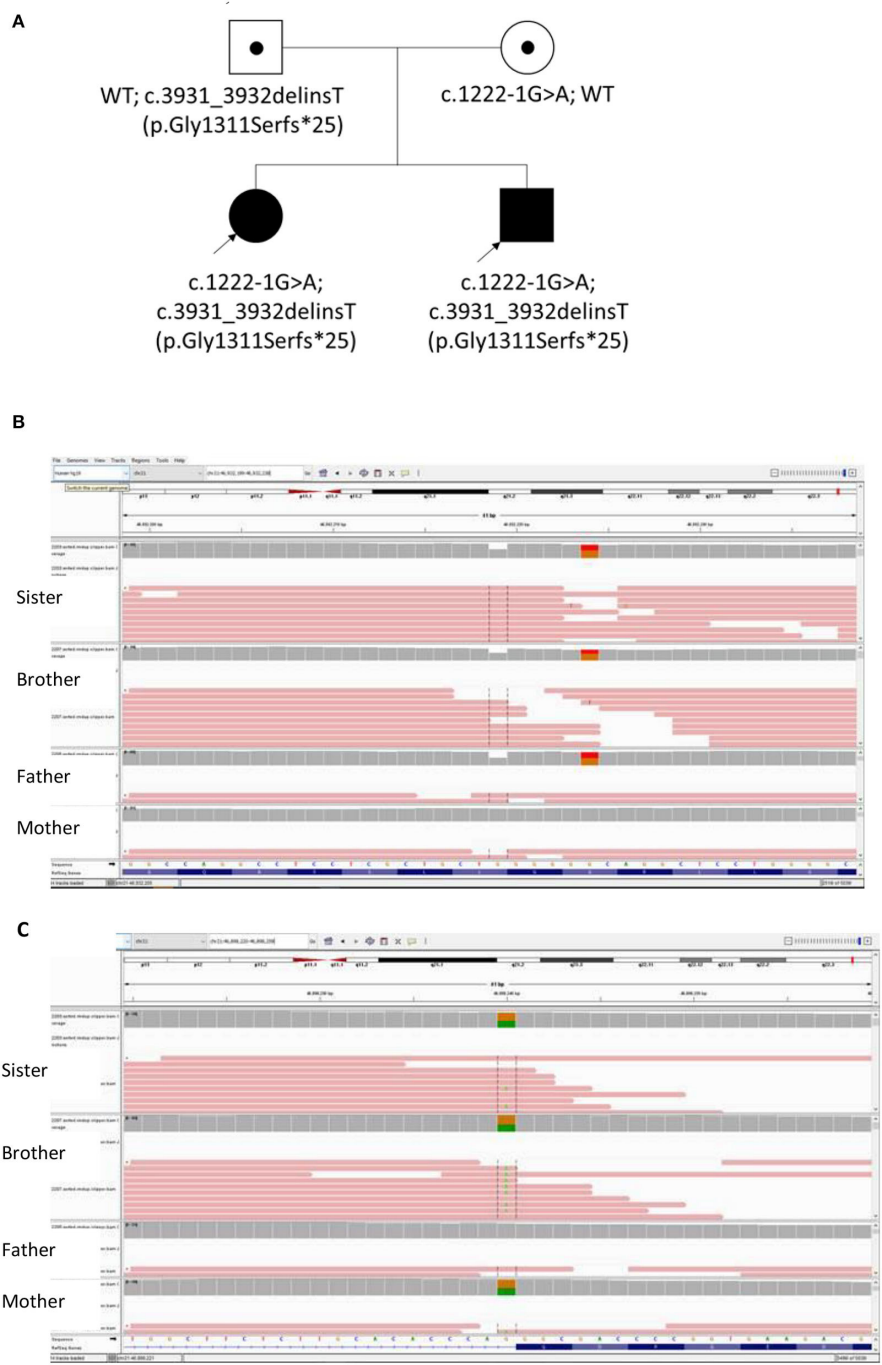
**(A)** The Pedigree of this Chinese family with biallelic changes in the *COL18A1* gene; **(B)** Integrated Genomics Viewer (IGV) screenshot of variant c.3931_3932delinsT inherited from father; **(C)** IGV screenshot of Variant 1222-1G>A inherited from mother.

Whole exome sequencing (WES) testing was performed for all four individuals of this family (Figure 2). Written informed consent was obtained from their parents. EDTA Blood sample from both patients and parents were taken. WES was performed through oligonucleotide-based target capture (SeqCap EZ MedExome, Roche) followed by next-generation sequencing (NextSeq500, Illumina). Sequencing reads are aligned to the Human Genome Assembly GRCh37/hg 19 using BWA. Variant calls are generated by the Genome Analysis Toolkit (GATK) HaplotypeCaller. The variants are then annotated and prioritized using ANNOVAR an in-house bioinformatics pipeline for SNP and small INDEL detection (v1 10830). Over 95% of reads were aligned to target. Over 99.3% target base were covered at >20X and the mean coverage of target bases was 102X. Variants were filtered for allele frequencies <1% in the Genome Aggregation Database (gnomAD) (https://gnomad.broadinstitute.org/) and basing on their types and genomic localization. Data interpretation is based on the understanding of genes and variants at the time of reporting. Considering the possible autosomal recessive pattern of inheritance, homozygous or compound heterozygous variants were more focused with analysis. With correlation of the patients' clinical phenotypes, compound heterozygous variants in the *COL18A1* gene (NM_001379500.1) were detected, including the maternally inherited variant c.1222-1G>A and the paternally inherited variant c.3931_3932delinsT p.(Gly1311Serfs^*^25) (Figure 2). Both variants have not been reported in general population or patients before. The c.1222-1G>A disrupts the canonical acceptor site of intron 8 and is predicted to cause aberrant splicing, resulting in an abnormal transcript that is subject to non-sense-mediated mRNA decay. Multiple *in silico* algorithms consistently predict the pathogenicity of this variant including SpliceAI delta score of acceptor loss as 0.99, CADD score as 32, and Human Splicing Finder (HSF) suggesting it mostly probably affect splicing. The c.3931_3932delinsT results in a reading frameshift on the last exon which changes a Glycine to a Serine at codon 1311, and creates a premature stop codon at position 25 of the new reading frame, possibly leading to a shorten protein. Both variants were classified as likely pathogenic according to the ACMG guideline (17, 18).

## Discussion

**Figure 3:**
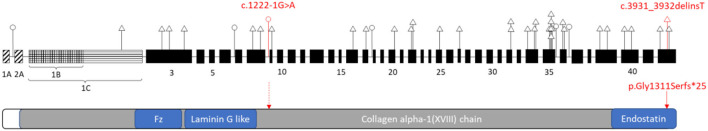
Schematic representation of the distribution of reported variants identified in *COL18A1* gene (top) related to the functional domains of the Type XVIII collagen (bottom). *COL18A1* gene (top): A, B, C indicated three isoforms: NM_001379500, NM_030582, NM_130444 respectively. The numbers indicated the exons. Triangle, variants in the coding region; Circle, variants in the non-coding region. Red labeled variants are the ones reported in this study. Please refer the other variant details to Supplementary Table 1.

We presented two novel loss-of-function variants in the *COL18A1* gene segregated in an affected Chinese family with Knobloch syndrome (KS). Currently majority of the *COL18A1* variants reported in the KS patients are frameshift, non-sense, and canonical splicing site changes causing loss of protein function (Figure 2). Unlike other collagenopatheis, mutations involving Glycine residues within the Gly-Xaa-Yaa repeats of the triple helix domain were not common causes of KS. The reported variants usually are unique for each patient or family which were more commonly in the C terminal of the protein and enriched in the Exon 34 and 35 (Figure 3).

This case report brings new genotype and phenotype information for KS. Firstly, KS could manifest antenatally with occipital encephalocele. Even if the encephalocele of KS presents early and with significant size, the condition could be managed surgically and the patient could have normal neurocognitive development as demonstrated by the two cases here. Currently no studies have found any correlation between the size or the severity of occipital abnormality and the site of the mutations in *COL18A1*. Khan et al. reported KS patients without any clinically discernible occipital defect (7). It would also suggest that early molecular diagnosis have important implications for management and counseling to these patients and their families.

Secondly, KS could show intra-familial variation in clinical presentations and severity. Only the younger brother showed hypoplasia of the inferior cerebellar vermis, dysgenesis of corpus callosum which were reported in other patients before (8). However, the brain malformations or neurological issue reported in the KS patients appeared with heterogeneity that may not be considered as core phenotype of KS (8, 19). When reviewing the details of these patients, they usually came from consanguineous families which other unrecognized genetic defects were associated with these uncommon presentations. The brother also showed more severe and early onset ocular abnormalities compared with the sister. It is important to follow up on the ophthalmological manifestation closely in the first year considering the retinal detachment could occur as early as 7 months of age.

Thirdly, defects in the *COL18A1* gene were reported to cause an autosomal dominant primary closed-angle glaucoma [MIM 618880] which was firstly observed in the parents or grandparents of KS-affected patients. Carriers in their mid-40 or younger usually were absent with angle closure indications suggested it was relatively late onset (20). Even though both parents in this family do not have relevant complaints currently, clinical follow-up for the parents or other obligate carriers in the extended family is suggested.

In conclusion, our report expand the genotype and clinical features in Knobloch syndrome with antenatal finding of occipital encephalocele and a normal neurocognitive development if interventions start early. Our patients received early neurosurgical intervention with resection and regular eye examination to ensure timely management. This illustrate early and regular ophthalmological examination could increase the chance of early therapy to the degeneration of retina or retinal detachment, which would be difficult to be noticed by parents.

## Data Availability Statement

The original contributions presented in the study are included in the article/Supplementary Files. The WES raw datasets of this family are not readily available due to ethical and privacy restrictions. Further inquiries can be directed to the corresponding author.

## Ethics Statement

The studies involving human participants were reviewed and approved by Ethics Committee of the Joint Chinese University of Hong Kong–New Territories East Cluster Clinical Research Ethics Committee. Written informed consent to participate in this study was provided by the participants' legal guardian.

## Author Contributions

SC, TL, and XZ designed the study. SC, Y-PY, EC, and XZ involved the patient management, collected samples, and followed-up. SC, Y-PY, YC, and S-SF performed the analysis and data interpretation. SC and YC wrote the manuscript. All authors contributed to the article and approved the submitted version.

## Conflict of Interest

The authors declare that the research was conducted in the absence of any commercial or financial relationships that could be construed as a potential conflict of interest.

## Publisher's Note

All claims expressed in this article are solely those of the authors and do not necessarily represent those of their affiliated organizations, or those of the publisher, the editors and the reviewers. Any product that may be evaluated in this article, or claim that may be made by its manufacturer, is not guaranteed or endorsed by the publisher.
