# Supplementary material for: Case Report: Novel Biallelic Variants in the COL18A1 Gene in a Chinese Family With Knobloch Syndrome
Source: Front Neurol. 2022 May 26;13:853918. doi: 10.3389/fneur.2022.853918 (PMC9178278; doi:10.3389/fneur.2022.853918)
Supplement: Supplementary file 1 [file Image_1.pdf]

Supp Figure 1 The fundi photography of the older sister showed degenerative retina with myopic changes and disc pallor.

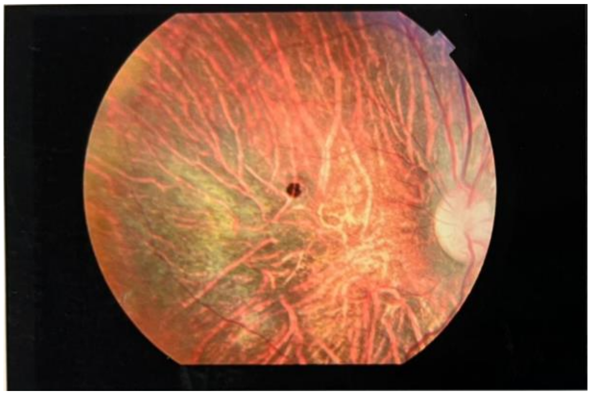

Right eye

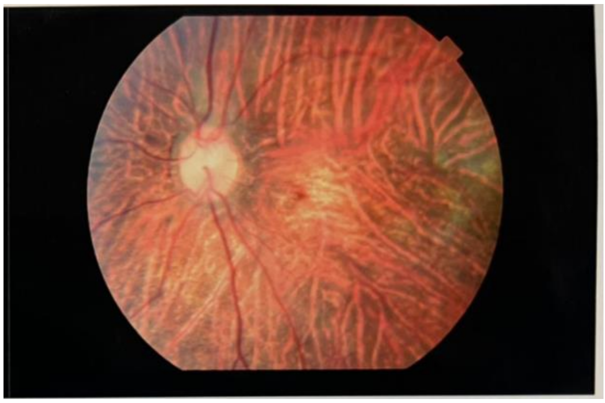

Left eye
